# Falling through the cracks: a qualitative study of HIV risks among women who use drugs and alcohol in Northeast India

**DOI:** 10.1186/1472-698X-13-9

**Published:** 2013-01-29

**Authors:** Michelle Kermode, Collins Z Sono, Chingzaning Hangzo Songput, Alexandra Devine

**Affiliations:** 1 Nossal Institute for Global Health, University of Melbourne, Level 4, 161 Barry St, Carlton, VIC, 3010, Australia; 2 Project ORCHID, Emmanuel Hospital Association, Dimapur, Nagaland, India; 3 Project ORCHID, Emmanuel Hospital Association, Imphal, Manipur, India

**Keywords:** Alcohol, HIV, India, Substance use, Women

## Abstract

**Background:**

HIV risks for women who inject drugs and those who engage in sex work are well documented. Women who are dependent on non-injecting drugs and alcohol are also likely to have increased vulnerability to HIV infection, but until they actually inject drugs or engage in sex work, are unlikely to come to the attention of HIV prevention programs.

**Methods:**

We undertook a qualitative study involving nine focus group discussions (FGDs) and 27 key informant interviews to investigate the context of female drug and alcohol use in two high HIV prevalence states of India (Manipur and Nagaland) and to describe their HIV risks. The FGD and interview transcripts were thematically analyzed

**Results:**

The women were relatively young (mean age 31 years in Manipur and 28 years in Nagaland), but 64% in Manipur and 35% in Nagaland were widowed or divorced. Both heroin and alcohol were commonly used by the women from Manipur, while alcohol was primarily used by the women from Nagaland, especially in the context of ‘booze joints’ (illicit bars). Reasons for drug and alcohol use included: to avoid symptoms of withdrawal, to suppress emotional pain, to overcome the shame of sex work, pleasure, and widowhood. HIV vulnerability was clearly described, not only in relation to injecting drug use and sex work, but also alcohol consumption.

**Conclusions:**

The contribution of alcohol use to the HIV vulnerability of women is not currently considered when HIV prevention programs are being designed and implemented leaving a group of high-risk women uncovered by much needed services such as treatment for a range of health problems including alcohol dependence.

## Background

Drug and alcohol use is a highly gendered behaviour in most parts of the world [1]. Gender differences have been observed in relation to the prevalence of substance use, initiation into use, patterns of use, reasons for use, and health and social consequences of substance use and dependence [1-7]. Women are more likely than men to: use drugs and alcohol as a consequence of difficult life circumstances and economic hardship; become dependent on substances; experience worse health consequences related to substance use; and engage in more HIV risk-behaviours in association with substance use [2,7,8].

### Substance use by women in India

Substance use has a long history in India. Drug use, especially injecting drug use, occurs mainly in large cities, and is particularly prevalent in the Northeast states of Manipur and, to a lesser extent, Nagaland. Alongside rapid economic growth in India, alcohol consumption has increased rapidly, although several states still prohibit the production, sale and consumption of alcohol [9-11]. Alcohol (such as rice beer/wine and spirits) is consumed mostly by men, and is widely available even in states where alcohol consumption is illicit.

The prevalence of substance use among women in India is not well documented, but an increase in female substance use has been anecdotally reported [2,12,13]. A large rapid assessment of substance users in fourteen Indian cities in 2000–01 found that 8% were women, and the substances they were using included heroin, alcohol and pharmaceutical products [2,3,8]. As is the case with men, women substance users tend to use more than one substance. In a large 2008 survey of 1258 women substance users from several states of India (including Manipur), 67% were current alcohol users (but only 6% used alcohol as their only substance), 25% were heroin users, 18% were dextropropoxyphene users, and 16% used sleeping pills [14].

### Substance use and HIV risks

The HIV risks for women who inject drugs and those who engage in sex work are well documented. Women who are dependent on non-injecting drugs and alcohol are also likely to have increased vulnerability to sexually transmitted infections (STIs) and HIV infection [3,15], but until they actually inject drugs or engage in sex work, are unlikely to come to the attention of HIV prevention programs.

Alcohol consumption potentially contributes to sexual disinhibition, sexual risk-taking, and compromised ability to negotiate and use condoms. There are links between alcohol use and sexual risk-taking [16-22], and between alcohol use and STIs and HIV infection [15,18,23-25]. Alcohol is often consumed by both sex workers and their clients for a variety of reasons including to heighten enjoyment, overcome shame, and gather courage to engage in commercial sex [21,26-28].

Although there is growing research interest in the links between alcohol use and HIV risks, including in India [10], many of the studies examine alcohol use by men [29-32], with the exception of a few studies that report on these links among FSWs [33,34]. A survey among 211 HIV positive FSWs in Mumbai found that 32% were heavy drinkers and 17% usually or always drank before having sex with a client. Although 90% of the women reported inconsistent condom use, this was not found to be associated with alcohol use. A qualitative study examining the use of alcohol in association with commercial sex in South India found that half of the FSWs avoided alcohol when having sex with a client because they wanted to stay in control in order to avoid difficult situations. Very little investigation has been done on alcohol use and HIV risks in the Northeastern part of the country.

### The context of Northeast India

Manipur and Nagaland are two Northeast Indian states that consistently report a high HIV prevalence, and in the case of Manipur, the highest in the country (adult HIV prevalence in 2009 was 1.4% in Manipur and 0.8% in Nagaland) [35]. The Northeast region is characterised by political unrest, deeply felt social conservatism, and substantial under-development. However, the states of Manipur and Nagaland are in many respects quite different from each other, not only socially, culturally and linguistically, but also in terms of the patterns of drug and alcohol consumption, and the nature of the HIV epidemic. Injecting drug use of heroin is far more common in Manipur, while the oral use and occasional injecting of pharmaceutical agents, especially Spasmoproxyvon (containing dextropropoxyphene), is more common in Nagaland. Heroin produced in the Golden Triangle region (Myanmar, Laos, Thailand) is smuggled into India and beyond through Manipur, with the result that the drug is readily available, which contributes to the high prevalence of injecting in this state. An estimated 1-2% of the adult population has injected drugs [36], with the result that injecting drug users (IDUs) have been the primary focus of HIV prevention interventions to date, and most IDUs are male. However, there is increasing recognition of the important contribution that sexual transmission is making to the spread of HIV in the region, especially in Nagaland, where HIV prevalence among IDUs in 2008 was only 3% (compared to 29% in neighbouring Manipur) (HIV Sentinel Surveillance 2008–09, personal communication, July 2010). In the same year, the prevalence among FSWs in Nagaland was 11%, and reported rates of syphilis among both IDUs and FSWs in Nagaland are also extremely high [37], indicating low levels of condom use, potential presence of an ulcerative STI, and increased risk for HIV transmission.

The sale and consumption of alcohol is illicit in both states, but relatively commonplace nevertheless. On the one hand there is strongly voiced moral disapproval of alcohol (as reflected in the state laws), but on the other hand alcohol is readily available and commonly consumed in an unregulated, illicit market. Many ‘booze joints’, that are essentially illicit bars, are visible across both states. As Benegal argues, these co-existing contradictory attitudes to alcohol predispose to ‘asocial behaviour, as well as chronic disabling alcoholism’ (p.1052) because there are no prescribed social norms to regulate people’s drinking behaviours [9].

The overall goal of the present study is to assess the HIV risks and health service needs of female (injecting and non-injecting) drug and alcohol users in two Northeast Indian states (Manipur and Nagaland) in order to promote improved access to services. This paper reports on findings in relation to the following objectives: 1. Understanding the local context of female drug and alcohol use in Manipur and Nagaland; and 2. Describing the HIV risks among the female drug and alcohol users. Findings related to other study objectives are reported elsewhere [38].

## Methods

### Study design

This qualitative study involved semi-structured, in-depth interviews with key informants (KIs) and focus group discussions (FGDs) with female drug and alcohol users in Manipur and Nagaland. The data collection took place in 2009–10. The study was facilitated by partnerships with local NGOs working in both urban and rural settings. These NGOs have existing relationships with female drug and alcohol users through their networks of outreach workers and peer educators. Local research officers (ROs) were trained and supervised by the study investigators to coordinate and conduct the data collection in each state.

A female drug or alcohol user was defined as a woman who judged herself to be a regular (injecting or non-injecting) user of one or more of the following drugs in the past six months: alcohol, heroin/brown sugar, propoxyphene/Spasmoproxyvon, cannabis or amphetamine type substances. All participants were aged ≥ 18 years.

### Data collection

#### In-depth interviews with key informants (KIs)

A total of 27 KIs were purposively recruited and interviewed (15 in Manipur and 12 in Nagaland). The KIs were from a range of government, private and NGO services, and included: directors, program managers and field workers from organizations that work with sex workers, drug users, and vulnerable women; workers from drug detoxification and rehabilitation centres; HIV testing counselors; nurses working with HIV patients; a journalist; and a ‘booze joint’ owner. Each interview took approximately one hour.

#### FGDs with drug and alcohol users

Five focus groups discussions (FGDs) were conducted with women alcohol and drug users in Manipur (2 in Imphal, 3 in Churachandpur), and four in Nagaland (3 in Dimapur, 1 in Wokha). There were 7–8 participants in each group, with a total of 39 participants in Manipur and 32 in Nagaland. Some groups included alcohol users exclusively, while others were a mixture of alcohol users, opiate users and those who used both substances. All FGDs were conducted by the local ROs, and each FGD lasted approximately two hours. FGD participants were identified through NGO outreach worker networks, and subsequent snowball sampling. Some of the participants were NGO service users and others were not, and while some were engaged in sex work, the majority were not. FGD participants were provided with payment to compensate for child care and travel costs.

Both the KI interviews and FGDs were conducted in the local language by the ROs using semi-structured interview guides that covered a range of thematic areas pertaining to female drug and alcohol users as listed below:

● Patterns of drug and alcohol use

● Learning to use drugs and alcohol

● Reasons for drug and alcohol use

● Commonly experienced problems

● Main health problems

● HIV risk behaviours

● Differences between women and men drug and alcohol users

● Types of services required

● Barriers and facilitators to service access.

The interview guides were developed with the literature and the study objectives in mind, then refined and piloted in collaboration with the Indian partner NGOs and local research team members. They were translated into the local languages (Paite, Manipuri and Nagamese) after detailed discussion of the intended meanings and appropriate language for each thematic area. All interviews and FGDs were digitally recorded, transcribed, and translated into English for subsequent analysis.

### Data analysis

The interview and FGD transcripts were thematically analyzed [39]. This involved systematically identifying and manually coding themes based on those covered in the interview guides. Following this initial coding of themes, sub-themes were inductively identified for each theme, and patterns and contradictions within and between themes and sub-themes elucidated using an iterative process. When undertaking the data analysis and reporting the findings, the subjective perspectives of the female drug and alcohol users were privileged over those of the KIs. However, the perspectives of the KIs tended to support and augment what the FGD participants had to say, and are also presented here.

### Ethical issues

All potential participants were informed about the nature and purpose of the study when they were invited to participate. Those who agreed to participate gave informed consent, and were assured of confidentiality. Ethics approval was obtained from the University of Melbourne Human Research Ethics Committee and the Institutional Review Board of the Emmanuel Hospital Association, New Delhi, India.

## Results

### Background information on participants

Demographic information of all FGD participants are summarized in Table 1. The proportion of widowed and divorced women was very high considering the relatively young age of the participants (64% in Manipur and 35% in Nagaland), highlighting the particular vulnerability of these women across India, including the Northeast.

**Table 1:** Demographic information for FGD participants

**Variable**	**Manipur n = 39**	**Nagaland n = 32**
**Mean age (range)**	31 yrs (20–45)	28 yrs(18–38)
- <25 yrs	26%	28%
- 25–29 yrs	15%	28%
- 30–34 yrs	21%	28%
- 35–39 yrs	33%	16%
- ≥40 yrs	5%	0
**Schooling**		
- None	18%	22%
- Not completed	61%	62%
- Completed	21%	16%
**Marital status**		
- Single	15%	34%
- Married	21%	31%
- Widowed	31%	13%
- Divorced	33%	22%
**Drug/alcohol use**		
- Alcohol	90%	91%
- Heroin	64%	0%
- Spasmoproxyvon	26%	16%
**Children**	64%	63%

### Drug and alcohol use among the women

The patterns of drug and alcohol use were different in each of the states. Most of the women from Nagaland were alcohol users, a small number used Spasmoproxyvon, and none were using heroin. The Nagaland study participants consistently reported that injecting drug use among women in their state was previously observed but is now very uncommon, and although women used Spasmoproxyvon orally, the most problematic substance for women in Nagaland was alcohol.

According to the study participants, even though Nagaland is a dry state, alcohol is widely available. Young women (as young as mid-teens) are employed in numerous booze joints that are commonplace, particularly in Dimapur (the commercial capital). These women are employed to attract male customers who are then encouraged to purchase alcohol not only for themselves but also for the young women, thereby increasing sales. Women drink mostly manufactured beer, locally brewed rice beer, and spirits. Over time some women become dependant on alcohol as indicated by the fact that they are drinking all day every day, and describe symptoms of alcohol withdrawal if not able to access alcohol.

"P: (participant): Sometimes even if we sell 8–9 cases of drinks, we still get a scolding from the owner of the joint… They tell us that we don’t have good communication skills, or we have not accompanied our customers well, and therefore to avoid this confrontation we force our customers to drink more and more by accompanying them, and we get drunk. (Focus Group Discussion (FGD)3 Dimapur, Nagaland)"

While the booze joints were the most frequently described context for alcohol consumption by women, some participants said that small groups of women congregate in drinking ‘hotspots’ or in the home of one of the women in order to drink. Additionally, some women, especially those who are married, drink alone at home where privacy can be maintained and social opprobrium avoided. However, this is only possible for women who have enough money to purchase a supply of alcohol to take home; many others are dependent on men in booze joints who purchase alcohol for them.

Alcohol use was similarly popular among the Manipuri women, but almost two-thirds were using heroin (No.4) and one-quarter Spasmoproxyvon. The women who used heroin tended to do so alone in quiet places, or at the peddler’s place. Most of the women using heroin injected it, but some were ‘chasing’ (inhaling) heroin.

There was widespread acknowledgement that women begin and continue to drink and take drugs for a complex range of reasons including poverty, family conflict, divorce or widowhood, pleasure, to deal with stress and suppress emotional pain, to overcome shyness and shame associated with sex work, and finally to avoid the symptoms of withdrawal.

"P: We drink and do drugs for different reasons. Some do so because of lots of stress in their lives, while some have a friend who drinks, and so she gives into peer pressure and goes astray… Some break off a serious relationship with their boyfriend and to ease the heartache, they get into drinking or do drugs. I am a married woman who looks after a booze joint, and so thinking of my [absent] children, I drink away, get drunk and then go to sleep. I wake up fresh [sober] and wash myself and then start drinking and go to sleep drunk. (FGD3 Dimapur, Nagaland)"

Table 2 provides examples of quotes that highlight the main reasons why women in Manipur and Nagaland use drugs and alcohol (as identified by the participants).

**Table 2:** Quotes reflecting some of the reasons women in Manipur and Nagaland use drugs and alcohol

**Reason**	**Quote**
**Widowhood**	Mainly, according to my experiences women who become widows at a very young age turn into alcoholic and female injecting drug users. I know 20–30 of them. Women who are very poor also become sex workers and start drinking alcohol… Mainly young widows, if they don’t have support from family, they start looking for ways of earning. If they do sex work then they compulsorily use alcohol or any type of drugs in order to avoid shyness and shame. (KI6 Manipur, Nurse)
**To suppress emotional pain**	My first husband eloped with me and later ran away with another girl. I got married again, and my second husband used to beat me up everyday and my face was always swollen. Sometimes, I couldn’t even open my eyes because of being beaten up. I left my second husband too. I drink because I cannot stop my tears from falling. I drink because I always cry (*broke down*). I feel so sad. Wherever I go, I go and drink lots of rum. Even before food, I drink a bottle of rum. Please don’t feel angry at me. (FGD3 Dimapur, Nagaland)
**For pleasure**	For me my friend taught me drinking alcohol while attending parties. Later on I started injecting heroin without being taught by anybody because I love to take drugs very much. It’s been almost seven years that I am injecting drugs. I was also in detoxification two times in between, but still I don’t want to stop, and so I continue. The moment I see the peddler’s house I want to take it. (FGD1 Imphal, Manipur)
**To avoid symptoms of withdrawal**	Now I am an addict. When I don’t take my daily dose, I feel lethargic and sick and stay in bed the whole day but whenever I have my daily dose, I feel light and active. (FGD1 Dimapur, Nagaland)
	I was selling drinks and in the process, customers asked me to drink with them and slowly I started drinking, and today I am an addict. If I don’t drink, I experience turkey [withdrawal]. From the time I get up, before I drink my morning tea, I take MC [rum] for breakfast (laughs). (FGD5 Wokha, Nagaland)
**To overcome shame associated with sex work**	For me there is nothing like sadness as the reason, but without drugs I cannot earn money, without this I cannot be together [have sex] with a man. With the drug I don’t feel shame. Like that I started drinking [alcohol]. (FGD2 Imphal, Manipur)

### HIV vulnerability

The women in this study were vulnerable to HIV infection in a range of ways that differed somewhat by state. For some of the participants, especially for those from Manipur who were heroin dependent, involvement in sex work was an obvious risk for sexual transmission of STIs including HIV. However, identifying as a sex worker also meant that they were able to access HIV prevention services that provided needles & syringes, condoms, and STI treatment. While some of the women indicated that they needed to engage in sex work in order to support their drug and alcohol use, it was also the case that some needed to use drugs and alcohol in order to engage in sex work.

The FGD participants from both states said that condoms were used some of the time, but not all the time. The failure to use condoms was sometimes because the men insisted on sex without a condom or paid extra money for it (in relation to sex work), but the intoxicated state of the woman was also commonly identified as a reason for condom-free sex, both in the context of sex work and outside of it.

"P1: Sometimes, men tell us that they will not use condoms even though we insist, and so sometimes we give in and do have sex without condoms. We also don’t know whether they are infected or not - they might be or they might not be… Men give us whatever amount of money we ask but on the grounds that we have sex without condoms. We do say no to them, but they get angry and tell us that they will not pay… We use sometimes, and sometimes we don’t. (FGD1 Dimapur, Nagaland)"

The risk of HIV infection secondary to injecting drug use tended to be a focus of the KIs more than the women, although some of the women who injected drugs acknowledged this possibility, and a few mentioned that they were HIV infected.

"P: These days as we can get free syringes from NGOs we use our own, and sharing of syringes is much less. However when we have severe withdrawal, as we do not always carry our own syringes we do not mind sharing, even if we know the HIV status of that person. This way it is easier for drug users [to be infected]. (FGD3 Churachandpur, Manipur)"

In both states, but particularly in Nagaland, the HIV risks for women dependent on alcohol were very evident. Many participants described situations that placed women alcohol users at high risk for infection with HIV. The consumption of alcohol by women was frequently linked to sex, mostly unprotected sex. Sometimes the women were raped when very drunk, sometimes by groups of men, and had limited recall for the event.

"P: As for me I started drinking after my husband died. I stayed with my brother who used to run a booze joint. Then whisky was known as bagpiper - it’s so strong - it was my first time. I drank this and got all drunk. I found myself naked in the morning after drinking. I didn’t remember anything about what had happened. My sister in law clothed me with mekhela [skirt]. She told me not to drink it [whisky] ever again… I started drinking beer and MC [rum]. We even go out with men without knowing how to put on a condom. Many of us women are like this. We are not even aware of whether we are going around naked or clothed. Most of the times we women drink because of problems faced with husbands. We even go looking for customers and have sex without condoms. (FGD3 Dimapur, Nagaland)"

"P1: It happens always. After we are drunk we don’t even care whether the guys we are with are sick or not. Sometimes we also find ourselves not properly clothed. I sleep at the booze joints."

"P2: Sometimes male friends group together and perform group sex with the girl. (FGD2 Dimapur, Nagaland)."

## Discussion

This qualitative study among women drug and alcohol users in two Northeast Indian states highlights the problem of both drug and alcohol use and dependence among women, and the very clear ways in which this places them at risk of HIV infection. While descriptions of HIV risks associated with drug use are not an unexpected finding, the strong focus given to the link between sexual risk behaviours and alcohol consumption in the context of relatively high HIV prevalence is an important finding that has implications for HIV prevention programs.

The findings also highlight the extent to which the women’s situations and issues varied by state. The women in Nagaland were mainly struggling with the consequences of alcohol use and dependence, and an absence of accessible services. Their HIV vulnerability was mostly related to the lack of control they had during sexual encounters due to their intoxicated state (and no doubt the intoxicated state of the men involved as well). The women in Manipur were also struggling somewhat with the consequences of alcohol use, but the most problematic substance in this state was heroin. Their HIV vulnerability was mainly associated with the need to frequently engage in sex work in order to raise money to purchase heroin. The observed differences between the two states could arguably be attributed to the non-representative sampling, but these differences are consistent with the experience of staff working in local HIV prevention and care services, and with other research [40].

The stated reasons for using drugs and alcohol overlapped with what has been reported elsewhere in the literature [14] and included widowhood, suppressing emotional pain, pleasure, avoiding the symptoms of withdrawal, and overcoming the shame of sex work. Even though the Northeast region of India is culturally, linguistically and ethnically distinct from the rest of the country, widowhood and divorce place women in situations of personal, social and financial hardship, similar to other parts of India. While some of these women may have been divorced due to their substance use, others were using substances to cope with being widowed or divorced.

Some of the women reported drinking alcohol primarily to avoid the symptoms of withdrawal. Unfortunately there are limited viable treatment options currently available for alcohol-dependent women. To the best of our knowledge, alcohol detoxification treatment for women is not available in either of these two states, and even if it were, many of the women would not have the money or family support required to access such a program. Similarly, there is no chapter of Alcoholics Anonymous for women in these states. Consequently, these women are effectively trapped in a cycle of alcohol dependence, which is challenging enough to recover from even when appropriate resources are available and accessible. Somewhat in contrast, those women who were dependent on injectable opioids such as heroin and Spasmoproxyvon have the option of registering to receive opioid substitution therapy (OST) as treatment for their opioid dependence, and there is at least one drug detoxification and rehabilitation centre that caters for women specifically. The literature indicates that women dependent on substances are less likely, over their lifetime, to enter treatment compared to men, but gender does not predict treatment retention, completion, or outcome if given the opportunity of treatment [41]. The participants in this study frequently emphasized the desperate need for women-only and women-friendly drug and alcohol detoxification and rehabilitation centres that are low cost and can accommodate children [38].

### HIV risks for female drug and alcohol users

The relationship between HIV risk behaviours and alcohol use has not received as much attention as the risks associated with injecting drug use and sex work [42], even though sex and alcohol use are frequently co-occurring behaviours all over the world. In a large study of women and substance use in India, Murthy noted that 60% of female substance users believed that substance use makes sex more enjoyable and less painful [14]. A survey among migrant FSWs in fourteen districts of four high HIV prevalence states (Andhra Pradesh, Karnataka, Tamil Nadu and Maharashtra) found that 54% consumed alcohol prior to sex, and that alcohol use was associated with inconsistent condom use [21]. Many of the FSW participants in our study said that they used alcohol in order to overcome the shame of engaging in sex work, and to cope with having sex with their clients. Thus it may be difficult for FSWs to reduce their alcohol use in the absence of alternative sources of income, and this has consequences for their ability to negotiate safe sex, and therefore their risk of HIV infection.

A link between alcohol use and unsafe sex is an important one, particularly in the state of Nagaland where the evidence suggests that sexual transmission of HIV is probably the major driver of the epidemic, which is somewhat different from the neighbouring state of Manipur where HIV transmission through unsafe injecting is still making a major contribution to the epidemic. Women who are injecting drug users can access a range of services through HIV prevention NGOs including STI management, needle & syringe and condom distribution, abscess management and basic health care. In contrast, women who are dependent on alcohol are not eligible for services provided by these NGOs unless they are engaging in sex work, as the NGOs are mostly funded to target HIV prevention services to specific high-risk groups (IDUs, FSWs, or men who have sex with men). It is likely that fear of discrimination because of their alcohol dependence would inhibit these women from accessing any mainstream health care services, even though many of them experience a range of health problems [38].

As alcohol is an illicit substance in Manipur and Nagaland, its consumption is inherently risky. The quality and serving of alcohol is totally unregulated, and venues that sell alcohol, while commonplace, are nevertheless trading outside of the law. Young women entering this world are vulnerable to a range of hazards including STIs, HIV, substance dependence, and violence [38]. While the (female) participants in this study provided vivid descriptions of unsafe sex linked to their own alcohol use, it is probable that alcohol use by men is also making a substantial and direct contribution to the occurrence of unsafe sex and sexual violence, and therefore a contribution to the prevalence of HIV and other STIs in these two states. Therefore, interventions to minimise the harms caused by alcohol use should target both women and men.

These study findings raise a number of key questions that need to be answered if the situation of women drug and alcohol users in Manipur and Nagaland is to be improved: How can HIV prevention NGO programs reach vulnerable women (non-injecting) drug users and alcohol users when they are only funded to target key population groups that do not generally include such women? If the NGO programs are not able to reach these women, who can/will? Women drug and alcohol users in India generally prefer to receive health care services through NGOs [14], so it would be beneficial if the HIV prevention NGO services currently offered to female IDUs and FSWs were extended to include other vulnerable women such as (non-injecting) drug users and alcohol users. This especially applies to safe sex promotion (including the distribution of condoms) and STI clinic services. Some of the participants mentioned that they were HIV infected, and it is highly likely that others are unknowingly HIV infected. The negative effects of alcohol on the progression of HIV disease are well recognised [43], so infected women need to be identified, offered treatment, and provided with relevant health promoting information.

This study has a number of limitations that should be considered when interpreting the findings. There could be some selection bias because the FGD participants were recruited through the NGO networks, but this is somewhat offset by the fact that the NGO workers deliberately recruited participants who were not recipients of their services. It is also possible that the women participating in the study are at the more chronic end of the drug and alcohol dependence spectrum, and therefore more conspicuous for recruitment into the study. Given the sensitive nature of the questions about socially taboo behaviours of women, some participants may have been inclined to provide more socially acceptable responses, at the expense of valid responses, resulting in bias. It is a qualitative investigation so findings cannot be generalized to all women drug and alcohol users in Manipur and Nagaland. A follow-up survey with a representatively sampled group of women would strengthen the findings.

## Conclusion

The findings from this study add to the growing body of literature linking risks for HIV infection with alcohol use, especially in the context of developing countries generally, and these two states of Northeast India in particular. Both the Manipur and Nagaland governments have embraced and successfully implemented a harm reduction approach to injecting drug use, and a similar approach to alcohol use and dependence is warranted. In particular, there is a real need for services to assist and provide treatment for women who are alcohol dependent. Many of the women in this study were at risk of STIs including HIV, and this is occurring in a context of relatively high HIV prevalence. HIV prevention efforts need to consider the vulnerability of women alcohol users, who are currently not covered by the HIV prevention services, if the HIV epidemic is to be comprehensively addressed.

## Competing interests

The authors declare that they have no competing interests.

## Authors’ contributions

All authors contributed to the conception and design of the study, CZS and CHS managed the data collection, MK and AD analysed the data, MK drafted the manuscript, all authors have reviewed the final manuscript.

## Pre-publication history

The pre-publication history for this paper can be accessed here:

http://www.biomedcentral.com/1472-698X/13/9/prepub
